# Network mechanisms of glymphatic system dysfunction in the disruption of the “brain-lung axis”

**DOI:** 10.3389/fneur.2026.1844527

**Published:** 2026-08-18

**Authors:** Lielong Mao, Zhiwei Yang, Feijun Jiang

**Affiliations:** 1 Department of Respiratory and Critical Care Medicine, The Second Hospital of Zhuzhou, Zhuzhou, Hunan, China; 2 Department of Medical Affairs, The Second Hospital of Zhuzhou, Zhuzhou, Hunan, China; 3 Department of Scientific Research, The Second Hospital of Zhuzhou, Zhuzhou, Hunan, China

**Keywords:** acute brain injury, acute lung injury, AQP4, brain-lung axis, glymphatic system

## Abstract

Acute brain injury (ABI) frequently precipitates severe extracranial target-organ complications, with the lungs being the most directly and fatally affected distant organs. Traditionally, the rapid onset of pulmonary dysfunction following brain injury has been attributed to the “massive catecholamine release”—a massive, dysregulated release of catecholamines leading to intense systemic vasoconstriction, elevated pulmonary capillary hydrostatic pressure, and subsequent neurogenic pulmonary edema. However, this purely neuro-hemodynamic model falls short of explaining the delayed onset and highly inflammatory nature of acute respiratory distress syndrome that persists during the later stages of injury. Recently, the discovery of the glymphatic system has provided a groundbreaking molecular and anatomical framework for understanding the pathological crosstalk within the “brain-lung axis.” As a macroscopic waste clearance network within the central nervous system highly dependent on the polarized expression of aquaporin-4 (AQP4), the glymphatic system undergoes structural and functional severe impairment following ABI. The loss of AQP4 polarization, coupled with reactive astrogliosis, halts cerebrospinal fluid-interstitial fluid exchange. This drainage failure not only forces the massive accumulation of damage-associated molecular patterns and pro-inflammatory cytokines within the brain parenchyma but also drives the systemic “spillover” of high-concentration neurogenic toxins through enzymatic disruption of the blood–brain barrier and the hijacked meningeal lymphatics. These brain-derived mediators—whether circulating freely or encapsulated within extracellular vesicles—travel via the systemic circulation to the pulmonary capillary bed. Upon reaching the lungs, they specifically target pulmonary microvascular endothelial cells by binding to TLR4 and RAGE receptors, triggering the phosphorylation and internalization of vascular endothelial cadherin, and thus completely dismantling the endothelial barrier. Concurrently, these signals drive the M1 polarization of alveolar macrophages, eliciting destructive neutrophil infiltration and parenchymal damage. Crucially, this pulmonary dysfunction generates severe hypoxemia and releases lung-derived inflammatory mediators that feed back to the central nervous system, establishing a fatal bidirectional vicious cycle. This narrative conceptual review aims to comprehensively dissect the bidirectional immune-inflammatory cascade network of the brain-lung axis triggered by glymphatic collapse and systematically evaluate the latest therapeutic prospects, considering the impact of pre-morbid systemic stressors and mechanical ventilation, while targeting AQP4 modulation, lymphangiogenesis, and systemic inflammation blockade.

## 1 Introduction

### 1.1 Evolution of the “brain-lung axis” concept: from massive catecholamine release to glymphatic breakdown

An intricate and bidirectional communication network exists between the CNS and the respiratory system, a trans-organ physiological and pathological interface widely termed the “brain-lung axis” (1, 2). For decades, the understanding of acute lung injury (ALI) secondary to severe brain injury within intensive care and neurosurgical settings was largely confined to the theory of autonomic hyperactivation (3). The classical paradigm postulated that severe cranial trauma or sudden spikes in intracranial pressure (ICP) trigger uncontrolled discharges in the hypothalamic and brainstem sympathetic centers, releasing massive quantities of epinephrine and norepinephrine (4, 5). This endocrine cataclysm, known as a “massive catecholamine release,” induces intense systemic vasoconstriction, shifting massive blood volumes into the lower-resistance pulmonary circulation (6). This abrupt surge overwhelms the hydrostatic pressure threshold of pulmonary capillaries, forcing protein-rich fluid into the alveolar space via mechanical “stress failure,” culminating in classic NPE (7, 8).

While the hydrostatic and massive catecholamine release theory elucidates the hyperacute pulmonary edema erupting within minutes to hours post-injury, it fails to explain the ARDS characterized by profound immune-inflammatory features that typically emerges 3 to 7 days post-ABI (9). Recent neurobiological breakthroughs have bridged this theoretical gap: the discovery of the glymphatic system and its functional impairment following brain injury marks a new era where molecular immunology intersects with neuroanatomy in brain-lung axis research (10, 11). The glymphatic system is not merely a physical clearance network maintaining the internal microenvironment of the brain; it serves as the central “floodgate” regulating the outward diffusion of central inflammation (12, 13). Recent reviews highlight that glymphatic dysfunction exerts systemic effects beyond the CNS, contributing directly to the breakdown of the brain-lung axis (14). When this system collapses post-injury, it metamorphoses from a protective clearance conduit into a focal source that amplifies and broadcasts neuroinflammation systemically, profoundly rewriting our fundamental understanding of brain-lung axis disruption.

### 1.2 High incidence and poor prognosis of ALI following ABI

In the clinical practice of the neurocritical care unit (NICU), pulmonary complications triggered by ABI (including severe TBI, high-grade aneurysmal SAH, and massive ischemic stroke) exhibit a striking incidence and exceptionally high mortality (15). Recent epidemiological meta-analyses and prospective cohort studies indicate that the incidence of ALI, progressing to severe ARDS, reaches 15 to 22% in patients with isolated severe TBI or high-grade SAH (16, 17). This secondary lung injury does not occur concurrently with the initial brain trauma; the median time to clinical diagnosis is typically around day 3 post-primary injury, which precisely coincides with the peak of intracerebral inflammatory cytokines and the most severe phase of glymphatic drainage stagnation.

Once an ABI patient develops ARDS, a fatal bidirectional vicious cycle is rapidly initiated. On one hand, extensive destruction of lung parenchyma and hyaline membrane formation lead to severe, refractory hypoxemia and hypercapnia, directly exacerbating the brain’s secondary metabolic crisis and inducing hypoxic–ischemic encephalopathy. Furthermore, the severely injured lungs no longer act merely as a passive target; they become an active secondary reservoir of systemic inflammation. Activated alveolar macrophages and injured pulmonary endothelium release massive lung-derived pro-inflammatory cytokines (such as IL-6 and TNF-*α*) and extracellular vesicles back into the systemic circulation. These lung-derived mediators travel back to the brain, infiltrating the already compromised blood–brain barrier to further aggravate neuroinflammation, exacerbate astrogliosis, and worsen glymphatic stagnation (18–20). On the other hand, mechanical ventilation strategies required to correct hypoxemia (e.g., high positive end-expiratory pressure, PEEP) impede jugular venous return. This elevated intrathoracic pressure not only further elevates ICP and causes a precipitous drop in cerebral perfusion pressure (CPP) (21), but critically, it mechanically suppresses normal CSF flow and glymphatic drainage, severely compounding the brain’s inability to clear neurotoxic waste.

### 1.3 Objective of the review

Given these critical clinical challenges, the primary objective of this review is to systematically delineate a network mechanism model based on the latest molecular and anatomical evidence, elucidating how the failure of the glymphatic system as the central “clearance pipeline” mediates targeted damage to the distal lung parenchyma. We will deeply deconstruct the microscopic dynamics of AQP4 polarization loss, trace the specific routes by which brain-derived toxic substances (especially DAMPs and extracellular vesicles) breach the BBB and utilize meningeal lymphatics to “spill over” into the systemic circulation, and uncover the molecular targets through which these mediators precisely attack the pulmonary vascular endothelial barrier and remodel the alveolar immune microenvironment. By bridging the mechanistic link from intracranial glial cells to pulmonary microvascular endothelial cells, this article aims to provide a panoramic cognitive framework for researchers and establish a robust theoretical foundation for future precision medicine strategies that salvage the “brain-lung axis” via targeted glymphatic protection. It is important to note that post-ABI ARDS is highly multifactorial; the glymphatic hypothesis operates in parallel with established clinical contributors such as ventilator-associated lung injury (VALI), secondary aspiration pneumonitis, and systemic fluid overload (22).

### 1.4 Literature search strategy

To comprehensively gather the latest evidence on the glymphatic system’s role in the brain-lung axis, a systematic literature search was conducted in PubMed and the Web of Science Core Collection, primarily targeting publications from the past decade. The search strategy utilized a combination of Medical Subject Headings (MeSH) and free-text keywords, employing Boolean operators (AND, OR) to intersect three core concept pools: central nervous system injury and drainage mechanisms (e.g., “Glymphatic System,” “Aquaporin 4,” “Meningeal lymphatics,” “Brain Injuries,” “Subarachnoid Hemorrhage”), distal pulmonary damage (e.g., “Acute Lung Injury,” “Respiratory Distress Syndrome,” “Neurogenic Pulmonary Edema”), and mediating molecular pathways (e.g., “DAMPs,” “HMGB1,” “Systemic Inflammation”). Crucially, during the literature selection process, original research was strictly prioritized.

## 2 Physiology and pathophysiology of the glymphatic system: microenvironmental catastrophe within the brain parenchyma

**Figure 1:**
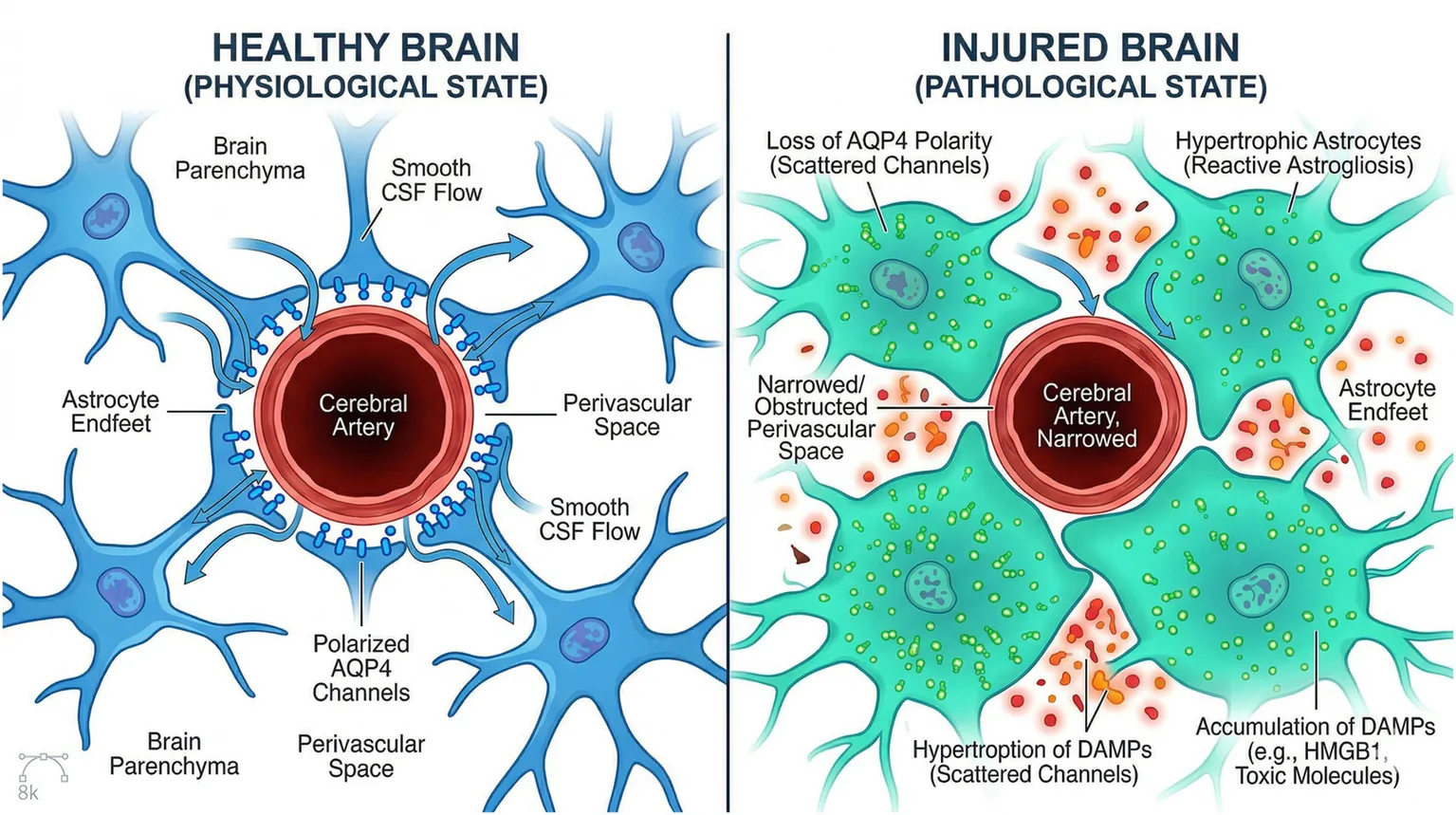
Physiological state and pathological disruption of the glymphatic system. Schematic comparison of the perivascular microenvironment. (Left) Under healthy physiological conditions, aquaporin-4 (AQP4) channels are highly polarized at astrocytic endfeet, tightly wrapping the penetrating cerebral arteries to facilitate efficient cerebrospinal fluid (CSF) influx and waste clearance. (Right) Following acute brain injury, reactive astrogliosis physically compresses the perivascular space. Concurrently, AQP4 channels lose their polarization and scatter across the astrocyte soma. This loss of structural and functional integrity halts fluid convection, leading to the severe extracellular accumulation of neurotoxic damage-associated molecular patterns (DAMPs), such as HMGB1.

From a neuroanatomical and neurophysiological perspective, the brain is the most metabolically active organ in the human body, yet it lacks a traditional intraparenchymal lymphatic vessel network (23). In its place is a highly specialized fluid convection and waste clearance network dependent on astrocytes and their perivascular endfeet—the glymphatic system (Figure 1).

### 2.1 AQP4-mediated CSF-ISF exchange under physiological conditions

In a healthy physiological state, the glymphatic system maintains fluid balance, nutrient distribution, and macroscopic metabolic waste clearance within the CNS (24). The anatomical basis of this system is the perivascular space (Virchow-Robin space), a continuous channel formed between the vascular smooth muscle layer of penetrating cerebral arteries and the overlying astrocytic endfeet (25). Cerebrospinal fluid (CSF) flows from the subarachnoid space down these periarterial spaces, penetrating deep into the brain parenchyma (26). The downward driving force for this fluid is primarily derived from the mechanical arterial pulsation generated by the cardiac cycle (27).

The core molecular cornerstone facilitating efficient exchange between CSF and interstitial fluid (ISF) is aquaporin-4 (AQP4). AQP4 is a specific channel protein mediating rapid transmembrane water transport. Under physiological conditions, its distribution in the brain exhibits high structural asymmetry, termed “polarization.” Over 80% of AQP4 is densely clustered on astrocytic endfeet directly abutting cerebral microvessels and the pia mater, forming orthogonal arrays of particles (OAPs), which are primarily mediated by the M1 and M23 isoforms of AQP4. This polarized distribution relies on the dystrophin-associated protein complex (DAPC), specifically *α*-syntrophin, which firmly anchors AQP4 to the perivascular basal lamina (28).

As arterial pulsation drives CSF into the periarterial space, assisted by low-resistance transmembrane water flow mediated by AQP4, CSF efficiently crosses the astrocytic endfeet, enters the brain parenchyma, and mixes with ISF. This mixed fluid creates a microscopic convective flow within the parenchyma, flushing toxic waste products generated by neuronal and glial metabolism (e.g., amyloid-*β*, p-Tau protein, and various cytokines) toward deep perivenous spaces (29, 30). Ultimately, this waste-laden fluid exits the brain parenchyma along the perivenous spaces, draining into meningeal lymphatics or the cervical lymphatic network. Notably, this physiological “washing” process is highly state-dependent; during deep non-rapid eye movement (NREM) sleep, a decline in central noradrenergic tone expands the extracellular space volume by approximately 60%, boosting CSF-ISF convective exchange efficiency to several times that of the awake state.

### 2.2 After ABI: loss of AQP4 polarization, astrogliosis, and drainage stagnation

Following ABI (such as severe TBI or SAH, primarily as a secondary cause of parenchymal ABI), this precise fluid dynamic and molecular anchoring system suffers devastating disruption, plunging the glymphatic drainage into complete stagnation. This systemic collapse originates from multidimensional pathophysiological cascades (31).

First, acute mechanical trauma (e.g., shear stress in TBI) or subarachnoid blood degradation products (e.g., fibrinogen and microthrombi in SAH) cause direct physical tearing of astrocytic endfeet and microcirculatory dysfunction (32–34). This severe physical and chemical stress triggers the disassembly of the DAPC anchoring complex (35). Stripped of its underlying molecular tether, AQP4 detaches from the perivascular endfeet and redistributes uniformly across the entire astrocytic soma (36). This critical mislocalization is termed “loss of AQP4 polarization” or AQP4 depolarization. The loss of AQP4 polarization severs the fluidic coupling between arterial pulsation and CSF parenchymal entry, severely reducing or completely halting directional trans-astrocytic water flow.

Second, ABI incites a robust neuroinflammatory response, inducing reactive astrogliosis. During the early to subacute phases of injury, astrocyte somas become markedly hypertrophic, accompanied by a sharp upregulation of marker proteins like glial fibrillary acidic protein (GFAP). The hypertrophic and proliferating astrocytes interweave to form a dense glial scar. While this structural remodeling partially isolates the injury core, its direct side effect is the physical compression and occlusion of the already narrow perivascular spaces. Consequently, molecular AQP4 depolarization and anatomical spatial occlusion caused by astrogliosis synergistically seal off the brain’s clearance pipeline, severely impairing the glymphatic system’s convective and clearance functions (37).

### 2.3 The consequences of drainage stagnation: cascade accumulation of DAMPs and cytokines

The most direct and destructive consequence of glymphatic stagnation is the entrapment of massive amounts of brain-derived toxic substances within the extracellular space (ECS). During ischemia, hypoxia, or physical tearing, necrotic or secondarily apoptotic neurons and glial cells release intracellular structural proteins, nucleic acids, and metabolites into the microenvironment, collectively known as DAMPs.

Key DAMPs driving the breakdown of the “brain-lung axis” include:

- *High mobility group box 1 (HMGB1)*: Normally a non-histone protein residing in the nucleus involved in DNA folding, HMGB1 transforms into a highly destructive pro-inflammatory alarmin once released extracellularly. It binds with high affinity to various pattern recognition receptors (PRRs, predominantly TLR4 and RAGE), initiating fierce inflammatory transcriptional cascades (38–40).
- *S100B protein*: A calcium-binding protein primarily secreted by astrocytes. Post-ABI, its local concentration rises exponentially. As a potent ligand for RAGE, it amplifies glial stress responses (41–43).
- *Neuron-specific enolase (NSE) and other metabolites*: These macromolecules continually accumulate in the undrained brain parenchyma (42, 44).

Due to the halted glymphatic flow, these high-concentration DAMPs cannot be transported to cervical lymph nodes for immune tolerization; instead, they continuously ferment within local brain tissues. By binding to TLR4 and RAGE receptors on microglia and astrocytes, they activate the MyD88 and NF-κB pathways, compelling resting microglia to rapidly polarize into the highly cytotoxic M1 (classically activated) phenotype (45, 46). M1 microglia subsequently unleash a deluge of primary pro-inflammatory cytokines, including interleukin-1β (IL-1β), interleukin-6 (IL-6), and tumor necrosis factor-*α* (TNF-α) (47, 48). This forms an uncontrollable death spiral: DAMP accumulation triggers an inflammatory storm, which further disrupts AQP4 polarization and exacerbates perivascular edema, ultimately turning the brain microenvironment into a highly invasive “pro-inflammatory microenvironment “capable of attacking the periphery.

## 3 “Spillover” and systemic propagation of brain-derived toxic substances: from cranial disaster to systemic alarm

**Figure 2:**
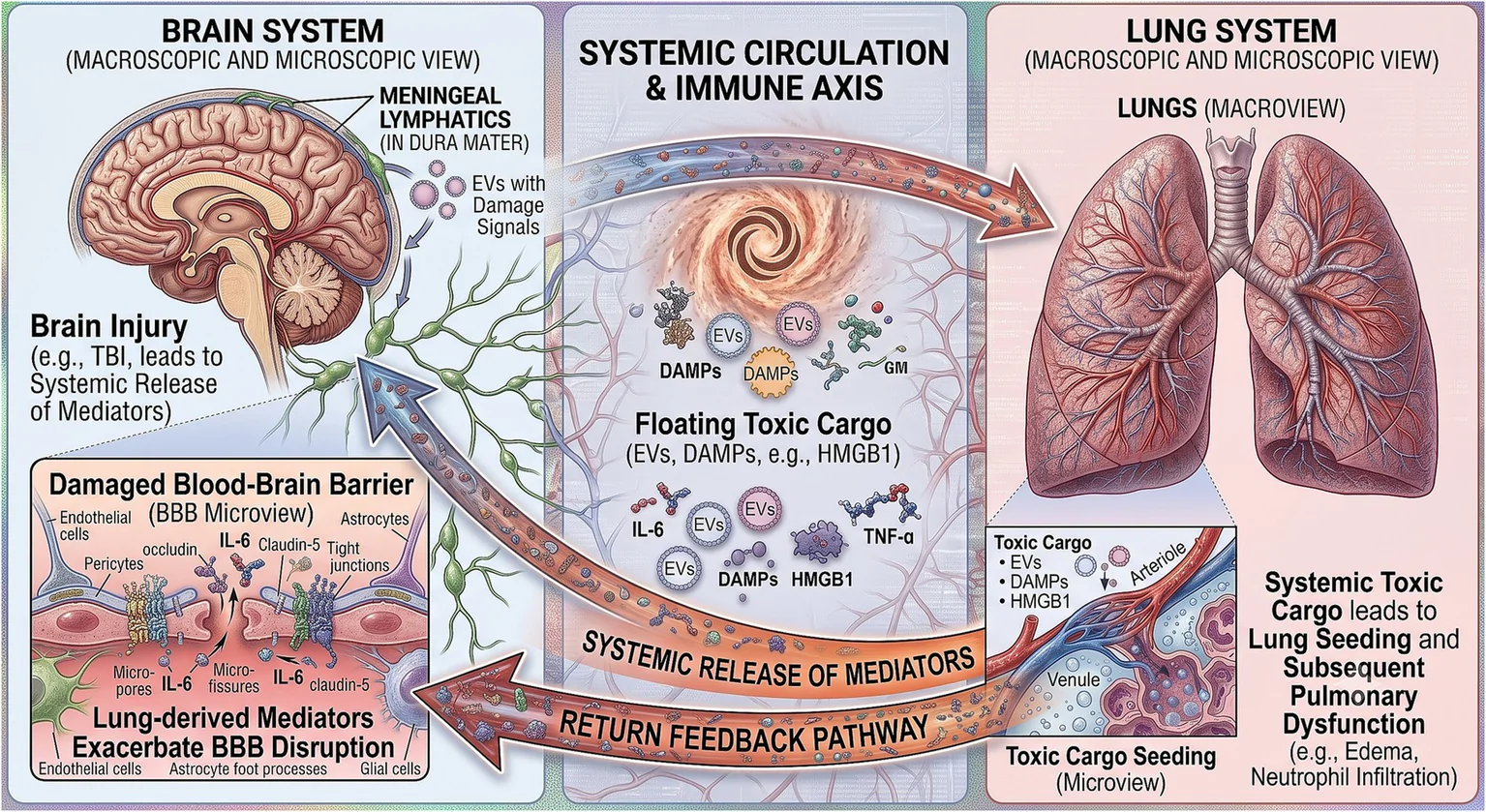
Systemic spillover and trans-organ propagation of brain-derived mediators along the brain-lung axis. Macroscopic and microscopic pathways of neuro-pulmonary crosstalk. Following severe brain injury, the enzymatic disruption of the BBB allows free DAMPs and brain-derived EVs carrying pathological microRNAs to leak directly into the systemic venous circulation. Simultaneously, hijacked meningeal lymphatic vessels drain high concentrations of inflammatory mediators into the deep cervical lymph nodes, priming peripheral immune cells. These neurogenic signals travel via the systemic circulation, ultimately depositing into the extensive pulmonary capillary network. Conversely, highlighting the bidirectional nature of the brain-lung axis, pulmonary damage induces severe hypoxemia and the release of lung-derived inflammatory cytokines and EVs into the systemic circulation. These secondary pulmonary insults feed back to the brain, exacerbating neuroinflammation, further disrupting the BBB, and creating a lethal bidirectional vicious cycle.

Within the closed, un-drained cranial vault, accumulating DAMPs and pro-inflammatory cytokines act as a rapidly pressurizing time bomb. The brain typically enjoys “immune privilege,” maintained by the strict filtration of the BBB and the organized drainage of meningeal lymphatics (49, 50). However, when severe neuroinflammation dismantles and remodels these anatomical defenses, intracranial toxins begin an uncontrolled “spillover” into the systemic circulation, initiating the systemic propagation phase of brain-lung axis collapse (Figure 2). The stagnation of glymphatic convective flow generates an extreme interstitial concentration gradient, physically driving these accumulated DAMPs across the enzymatically compromised BBB into the systemic circulation.

### 3.1 Enzymatic disruption of the BBB and the dual role of meningeal lymphatics

The mechanical and enzymatic degradation of the BBB serves as the primary gateway for brain-derived toxic substances to flood into the peripheral blood. M1 microglia and reactive astrocytes, activated by DAMPs and local cytokines (like IL-1β and TNF-*α*), synthesize and secrete massive quantities of matrix metalloproteinases (MMPs, especially MMP-3 and MMP-9). Acting as molecular scissors, these proteolytic enzymes specifically degrade key structural proteins maintaining endothelial tight junctions, such as Claudin-5, Occludin, and Zonula Occludens-1 (ZO-1). Accompanied by endothelial cytoskeletal contraction and pathological widening of intercellular gaps, BBB permeability increases drastically. Previously confined gigatons of HMGB1, S100B, and free cytokines leak directly down their concentration gradients into capillary and venule networks, rapidly merging into the systemic venous circulation.

Concurrently, recent advances in neuroimmunology have uncovered the complex “dual role” played by meningeal lymphatic vessels (mLVs) located in the dura mater during this pathological process (51). Under physiological conditions or mild trauma, mLVs exert protective clearance and immune surveillance functions, absorbing antigen-laden CSF and draining it orderly toward the skull base to eventually reach deep cervical lymph nodes (dCLNs) (52). However, during severe ABI and glymphatic dysfunction, the extreme burden of inflammatory mediators and tissue debris can physically obstruct or paralyze mLVs due to lymphatic endothelial damage, plunging immune surveillance efficiency. More lethally, the lymphatic vessels that remain patent transform from benign antigen-presenting channels into a high-capacity “toxin highway.” Ultra-high concentrations of brain-derived cytokines and DAMPs march straight through the mLVs to the dCLNs, causing aggressive priming of the peripheral immune system. Here, antigen-presenting cells present brain-derived antigens to peripheral T cells and monocytes, inducing systemic immune dysregulation. This mobilizes peripheral immune cells to be released masse into the systemic circulation, providing ample “ammunition” for the impending inflammatory storm in the lungs.

### 3.2 Tracking systemic propagation and “stealth delivery” via EVs

After breaching anatomical barriers, these concentrated brain-derived toxic mediators must traverse the systemic circulation to reach the lungs. From an anatomical-dynamic perspective, toxic substances entering capillaries via the damaged BBB or joining the venous angles via cervical lymph nodes directly converge into the internal jugular vein, subsequently entering the superior vena cava. Because the pulmonary circulation is the first—and the most expansive (over 100 square meters of surface area)—microvascular bed encountered by all returning venous blood, the lungs inevitably become the “first filter” and the most direct target organ for brain-derived toxins.

Notably, recent molecular investigations reveal that this propagation is not solely reliant on the free diffusion of molecules in the blood. A significant proportion of highly pathogenic mediators are encapsulated within brain-derived EVs for targeted delivery (53). EVs are nanoscale lipid-bilayer vesicles (typically 30–150 nm in diameter) secreted by damaged neurons, astrocytes, and microglia (54). Owing to their lipid bilayer structure, EVs possess formidable penetrating ability, easily crossing the disrupted BBB while shielding vulnerable encapsulated macromolecules (e.g., proteins, RNAs) from degradation by abundant blood proteases and nucleases (55).

Post-ABI, not only does the quantity of brain-derived EVs explode, but their “cargo” also undergoes significant pathological alteration. Studies demonstrate that EVs are a crucial, stealthy vehicle for the release and systemic dissemination of DAMPs like HMGB1 and inflammasome components. Crucially, these EVs are enriched with pathological microRNAs (miRNAs) wielding epigenetic regulatory functions, such as miR-362 (56). When these EVs, carrying the “codes of neural injury, “reach the pulmonary circulation, they are directly taken up by pulmonary microvascular endothelial cells and alveolar macrophages via membrane fusion or receptor-mediated endocytosis. This precisely unleashes the brain’s “destruction directives” into the cytoplasm of lung cells, triggering deep gene expression reprogramming and targeted attacks.

## 4 Targeted attack on the lungs: from endothelial barrier disruption to immune microenvironment remodeling

**Figure 3:**
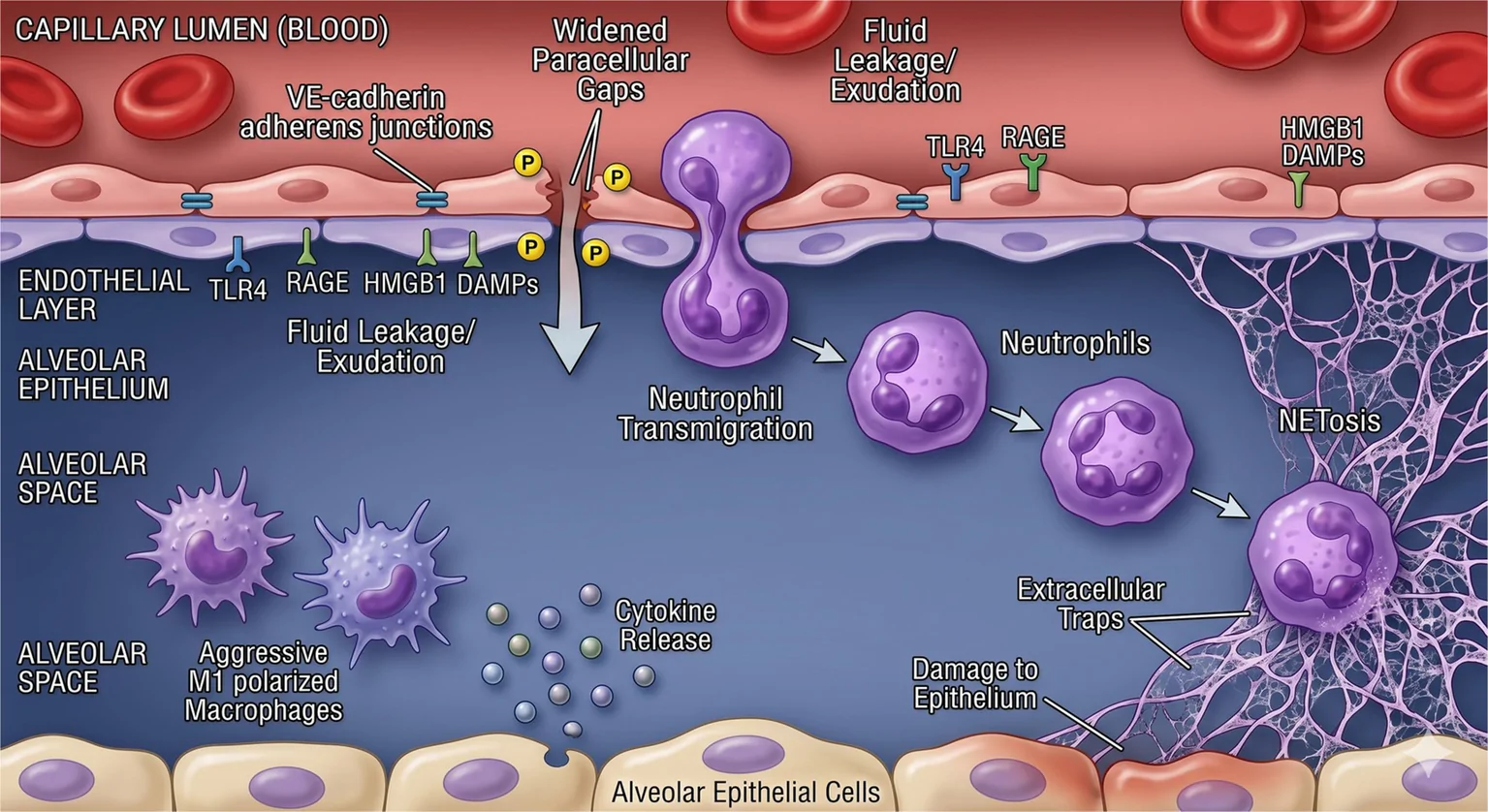
Endothelial barrier disruption and immune remodeling in the pulmonary microvasculature. Microscopic view of the alveolar-capillary barrier breakdown. Within the capillary lumen, brain-derived HMGB1 binds to TLR4 and RAGE receptors on pulmonary endothelial cells, triggering rapid kinase signaling that causes VE-cadherin phosphorylation and internalization. This dismantling of adherens junctions forms paracellular gaps, leading to severe protein-rich fluid extravasation. In the alveolar space, these inflammatory signals drive the M1 polarization of alveolar macrophages and recruit neutrophils, which subsequently undergo NETosis, releasing destructive neutrophil extracellular traps (NETs) that inflict lethal damage on alveolar epithelial cells.

As free serum proteins laden with HMGB1 and S100B, primed peripheral immune cells, and brain-derived EVs carrying pathological miRNAs surge into the pulmonary capillary bed, the structural devastation of the lungs officially commences (57). This process exhibits astonishing molecular specificity, progressing via two main pathways: the direct destruction of the vascular endothelial physical barrier, and the malignant remodeling of the alveolar immune microenvironment (Figure 3). Because the intracranial glymphatic clearance remains severely impaired, the brain acts as a continuous upstream generator of EVs and DAMPs, actively preventing the resolution of pulmonary inflammation. However, it is crucial to recognize that this communication is not a unidirectional downstream cascade. Once the pulmonary immune microenvironment is mal-remodelled, the lungs transition from a ‘victim’ organ to a potent secondary driver of pathology. The intense oxidative stress, trapped neutrophils, and alveolar damage generate a massive influx of retrograde inflammatory signals (hypoxemia, lung-derived DAMPs) back to the central nervous system. This bidirectional feedback severely compounds neural vulnerability, creating an interdependent cycle of neuro-pulmonary deterioration where neither organ can resolve its inflammation without the other.

### 4.1 Brain-derived DAMPs/cytokines bind TLR4, dismantling the VE-cadherin endothelial barrier

The integrity of the alveolar-capillary barrier is a prerequisite for maintaining normal gas exchange and preventing fluid extravasation. The core stability of this physical barrier relies on the adherens junctions (AJs) between pulmonary microvascular endothelial cells, with Vascular Endothelial Cadherin (VE-cadherin) acting as the most critical transmembrane structural protein (58).

The luminal surface of pulmonary vascular endothelial cells constitutively expresses high levels of various pattern recognition receptors (PRRs), particularly TLR4 and RAGE. When brain-derived DAMPs (such as HMGB1) arrive at the pulmonary capillaries, they exhibit extremely high affinity for TLR4 and RAGE, executing specific binding. The activation of the HMGB1-TLR4/RAGE axis not only triggers classical MyD88-dependent pathways—leading to NF-κB nuclear translocation and localized secondary inflammatory cascades—but critically, it rapidly initiates rapid kinase signaling pathways that dismantle endothelial junctions.

Upon receptor activation, intracellular pro-permeability signaling molecules, such as c-Src kinase and RhoA-associated coiled-coil forming protein kinase (ROCK), are swiftly activated, simultaneously inhibiting vascular endothelial protein tyrosine phosphatase (VE-PTP), which normally maintains barrier tightness (59). This signaling imbalance leads to hyperphosphorylation of key tyrosine residues in the intracellular domain of VE-cadherin. Phosphorylated VE-cadherin not only loses physical binding affinity with its intracellular anchoring proteins (like p120-catenin and plakoglobin) but also undergoes rapid internalization from the cell membrane, entering intracellular degradation pathways. The downregulation and internalization of VE-cadherin forcefully “unzip” the adherens junctions between adjacent endothelial cells, forming paracellular gaps. This total collapse of endothelial connectivity causes pulmonary capillary permeability to skyrocket exponentially, allowing protein-rich fluid, red blood cells, and activated immune cells to pour unrestrictedly into the pulmonary interstitium and alveolar spaces, yielding the hallmark protein-rich exudate and severe pulmonary edema of ARDS.

### 4.2 M1 polarization of alveolar macrophages and destructive neutrophil infiltration

Beyond the collapse of physical barriers, the pulmonary immune microenvironment undergoes profound rearrangement. Under physiological conditions, alveolar macrophages (AMs) residing on the alveolar surface maintain a resting (M0) or reparative (M2) polarization state, possessing high phagocytic capacity without inciting severe inflammation to preserve immune tolerance. However, when exposed to the barrage of brain-derived EVs and DAMPs (e.g., high levels of HMGB1, ATP) that flood the lungs secondary to BBB disruption, these macrophages are forced to undergo abrupt phenotypic switching, polarizing towards the classically activated, pro-inflammatory M1 phenotype.

Once polarized to M1, alveolar macrophages act like highly reactive effector cells. NADPH oxidases (such as the NOX2 system) on their cell membranes are strongly activated, sparking an explosive generation of reactive oxygen species (ROS) and creating an environment of intense oxidative stress. Simultaneously, these M1 cells unleash floods of chemokines (e.g., CXCL1, IL-8) and primary pro-inflammatory cytokines (IL-1β, TNF-*α*) (60). Furthermore, bone marrow-derived monocytes and neutrophils—mobilized by brain injury inflammatory signals—massively roll, adhere, and transmigrate across the already VE-cadherin-depleted endothelial gaps, surging aggressively into the lung interstitium and alveolar spaces under the guidance of CXCL1 chemotactic gradients.

Upon entering the alveolar space, neutrophils degranulate under extreme inflammatory stimulation, releasing neutrophil elastase and matrix metalloproteinases that further degrade alveolar epithelial cells. More, driven by HMGB1 and severe oxidative stress, large numbers of neutrophils undergo NETosis, extruding neutrophil extracellular traps (NETs) composed of web-like DNA structures and microbicidal proteins (61). Although NETs originally evolved to ensnare invading pathogens, in this scenario of sterile neuroinflammation, widespread NETs directly inflict lethal damage on fragile type I alveolar epithelial cells and surfactant-secreting type II alveolar epithelial cells, resulting in alveolar collapse and the blockade of normal gas exchange.

### 4.3 Molecular distinction between NPE and typical ARDS

While NPE and brain-injury-associated ARDS both present with diffuse bilateral pulmonary infiltrates and severe hypoxemia on clinical imaging, their underlying pathobiology and molecular drivers are fundamentally distinct. To delineate this temporal and pathological transition, the table below systematically contrasts classic sympathetic-driven NPE with ARDS driven by glymphatic dysfunction (Table 1).

**Table 1:** Comparison of mechanistic and molecular features between neurogenic pulmonary edema (NPE) and brain-derived ARDS mediated by glymphatic dysfunction.

Clinical and molecular features	Neurogenic pulmonary edema (NPE)	ABI-associated ARDS (glymphatic dysfunction-driven)
Time of onset	Hyperacute phase (minutes to hours post-primary injury)	Delayed/Subacute phase (median onset generally 3 to 7 days post-injury)
Core driving mechanism	Central sympathetic storm, massive catecholamine release	Glymphatic clearance failure, DAMP spillover, and systemic inflammatory amplification
Hemodynamic changes	Intense systemic and pulmonary vasoconstriction; transient spike in pulmonary capillary hydrostatic pressure	Inflammatory vasodilation; progressive, catastrophic increase in pulmonary vascular endothelial permeability
Capillary pathology	Predominantly mechanical “Stress failure”	VE-cadherin internalization/degradation; enzymatic cleavage of tight and adherens junctions
Plasma/lung fluid markers	Rapid elevation of epinephrine, norepinephrine, neuropeptide Y	High levels of DAMPs (HMGB1, S100B, NSE), elevated IL-6, TNF-*α*, vWF
Extracellular vesicles (EVs)	Lack of specific pro-inflammatory EV enrichment	Massive influx of pathological brain-derived EVs carrying miR-210, miR-338-3p, and ACE+ EVs
Alveolar immune response	Mild early stress changes; lack of significant parenchymal destruction	Widespread M1 macrophage polarization; massive neutrophil infiltration, degranulation, and NETosis
Edema fluid nature	Transudate: low protein content, few cellular components	Exudate: High protein content, rich in cellular debris, fibrin, and inflammatory cells

### 4.4 The afferent loop: pulmonary dysfunction exacerbates neural vulnerability

Crucially, the brain-lung crosstalk is not unidirectional but constitutes a pathological bidirectional vicious cycle. As pulmonary pathologies such as ARDS develop, they profoundly impact neural vulnerability. The resulting refractory hypoxemia and hypercapnia directly induce cerebral vasodilation, elevating intracranial pressure (ICP) and compounding the brain’s metabolic crisis (62, 63). Furthermore, the injured lungs act as a secondary inflammatory reservoir. Activated alveolar macrophages and injured pulmonary endothelium release massive quantities of systemic cytokines (e.g., IL-6, TNF-*α*) and lung-derived EVs. These peripheral inflammatory signals breach the already compromised BBB, triggering autonomic neuro-dysregulation and an intensified immune feedback loop within the CNS. This secondary hit exacerbates reactive astrogliosis, further polarizing microglial activation, and continuously suppresses glymphatic outflow, thereby trapping the patient in a lethal feedback loop of interdependent organ failure (16, 64, 65).

## 5 Therapeutic prospects targeting the glymphatic system to protect the “brain-lung axis”

Given that pulmonary complications following ABI are deeply rooted in the physical severe impairment of the intracranial glymphatic system and the subsequent systemic “spillover” of immune-inflammation, the focus of modern neurocritical intervention is undergoing an unprecedented paradigm shift. The frontier of clinical treatment is pivoting from isolated pulmonary support (e.g., mechanical ventilation adjustments) to disrupting the malignant axis of trans-organ communication at its source. Strategies to protect the “brain-lung axis” primarily focus on two core dimensions: repairing glymphatic clearance physically or pharmacologically within the cranium, and precisely intercepting the spread of neurogenic inflammatory cascades in the systemic circulation.

### 5.1 Pre-morbid vulnerabilities and mechanical ventilation as modulators

When evaluating therapeutic potential, it is imperative to recognize that therapeutic frameworks cannot treat the organs merely as isolated **“**sources**”** or “victims,” but rather as interdependent victims within a bidirectional crosstalk. Furthermore, the efficacy of any intervention is heavily influenced by pre-morbid systemic stressors and environmental modulators. Patients with pre-morbid obstructive sleep apnoea (OSA) suffer from chronic intermittent hypoxia, which severely disrupts the circadian control of the glymphatic system. Similarly, environmental factors like cigarette smoke and ozone in chronic obstructive pulmonary disease (COPD) chronically exacerbate oxidative stress and influence DAMPs. These pre-morbid conditions induce chronic glymphatic outflow dysfunction, alter lung-to-brain EV signaling, and disrupt the peri-plaque microenvironment, often leading to amyloid-beta accumulation and astrocyte dysfunction even prior to the acute ABI (66–68). Consequently, when ABI occurs, these patients possess a highly vulnerable neuro-immune-pulmonary network. Additionally, the inevitable clinical use of mechanical ventilation limits therapeutic efficacy by artificially increasing ICP and physically suppressing CSF flow, necessitating highly personalized ventilation strategies that balance oxygenation with glymphatic patency.

### 5.2 Pharmacological and physiological interventions to improve glymphatic function

The key to reversing glymphatic stagnation lies in restoring the fluid dynamics of the brain interstitial space and repairing AQP4 spatial polarization, thereby unblocking the macromolecular “clearance pipeline.”

#### 5.2.1 Sleep regulation and the neuroprotective role of dexmedetomidine

The fluid convection efficiency of the glymphatic system is profoundly regulated by the sleep–wake cycle. During slow-wave sleep in the deep NREM phase, central noradrenergic tone drops significantly, expanding perivascular space volume and minimizing interstitial fluid drainage resistance, thereby maximizing the clearance efficiency of amyloid proteins and DAMPs. In states of severe brain trauma or postoperative stress, patients often experience profound sleep deprivation and neurotransmitter disruption, further exacerbating the severe impairment of the clearance system.

*Dexmedetomidine (Dex)*, a highly selective α2-adrenergic receptor agonist, has demonstrated remarkable therapeutic potential in this context. At the molecular and network level, Dex specifically inhibits locus coeruleus-mediated norepinephrine release, inducing an electrophysiological pattern highly analogous to natural slow-wave sleep (69). By simulating deep sleep, Dex effectively expands the physical spaces between astrocytes, boosting CSF flow. Critically, preclinical studies confirm that Dex significantly stabilizes BBB tight junction proteins (including Claudin-5 and Occludin) and effectively prevents AQP4 dislocation from astrocytic endfeet caused by surgical stress or trauma (70). By suppressing local neuroinflammation and restoring AQP4 polarization, Dex reignites the glymphatic convection mechanism, promptly flushing harmful metabolites from damaged brain regions, thereby cutting off the accumulation and peripheral spillover of inflammatory factors at the source (71).

#### 5.2.2 Phased intervention strategies using AQP4 modulators

Given the central role of AQP4 in brain fluid transport and water balance and convective transport, direct pharmacological regulation via AQP4 Modulators has emerged as a highly attractive target. Representative drugs such as TGN-020 and AER-271 (and its prodrug AER-270) are currently the most extensively studied selective AQP4 inhibitors (72, 73).

During the hyperacute phase (within hours) of ischemic stroke or TBI, astrocytes rapidly absorb water via AQP4, leading to cytotoxic edema that mechanically compresses and occludes perivascular spaces. Administering AQP4 inhibitors like TGN-020 or AER-271 at this stage effectively blocks water influx, significantly alleviating cerebral edema, preventing the irreversible expansion of the ischemic core, and maintaining initial BBB integrity. However, because AQP4-mediated transmembrane water transport is equally vital for maintaining glymphatic convection and waste clearance during the middle-to-late injury stages, prolonged blanket inhibition can paradoxically result in protein waste accumulation (74). Thus, future applications of AQP4 modulators will trend toward precise “phased therapies”: utilizing small-molecule inhibitors to resist edema in the acute phase, while employing gene therapies or nanoparticle drugs in the subacute phase to target the DAPC complex (e.g., *α*-syntrophin), chemically re-anchoring AQP4 channels to restore polarized perivascular expression and re-initiate robust CSF-ISF convective flushing.

#### 5.2.3 Pro-lymphangiogenic therapy for mLVs

To address congestion at the downstream exit of the glymphatic system, enhancing the drainage capacity of meningeal lymphatics toward cervical lymph nodes represents another cutting-edge approach. Recent studies demonstrate that adeno-associated virus (AAV)-mediated delivery or local hydrogel administration of Vascular Endothelial Growth Factor C (VEGF-C) post-injury robustly stimulates the proliferation and dilation of dural lymphatic vessels (lymphangiogenesis) (75). The application of VEGF-C significantly increases the number of lymphatic branches and vessel diameters, vastly improving the drainage efficiency of CSF and DAMP-rich interstitial fluid (76, 77). This “sewer-widening” strategy accelerates CCL2-dependent macrophage debris clearance and lowers intracranial pressure, simultaneously alleviating the burden of dumping toxic substances directly into the blood circulation. This dual effect protects the brain while effectively shielding the lungs from distal injury.

### 5.3 Blocking systemic inflammatory cascades: intercepting brain-derived “inflammatory mediators “

When glymphatic severe impairment is established and brain-derived toxins have breached the BBB in large quantities into the systemic circulation, the final line of defense for the lungs is to intercept these lethal signals directly within the peripheral blood or the local pulmonary microvasculature.

Among all brain-derived DAMPs, HMGB1 stands as the primary therapeutic target due to its extreme destructive potency and broad receptor-binding capacity. The systemic administration of anti-HMGB1 neutralizing monoclonal antibodies (Anti-HMGB1 mAbs) has demonstrated game-changing efficacy in various *in vivo* animal models of severe TBI and stroke complicated by lung injury. Once injected into the bloodstream, these antibodies rapidly bind and neutralize freely circulating HMGB1 as well as HMGB1 complexes released via EVs. By establishing a blockade at the ligand level, the antibodies effectively prevent HMGB1 from contacting TLR4 and RAGE receptors on the surface of pulmonary capillary endothelial cells.

This precise targeted blockade directly short-circuits the secondary MyD88 and NF-κB-mediated inflammatory cascades within the lungs, successfully averting the phosphorylation and subsequent internalization and degradation of VE-cadherin by ROCK and c-Src kinases. Consequently, adherens junctions between endothelial cells remain structurally intact, vascular permeability is normalized, and the exudation of protein-rich fluid is fundamentally prevented. Furthermore, intercepting HMGB1 deprives alveolar macrophages of their core driver for M1 polarization, significantly reducing the secretion of neutrophil chemokines and halting the occurrence of interstitial NETosis and extensive epithelial necrosis. Similarly, pharmacological inhibitors of HMGB1 like Glycyrrhizin, or specific TLR4 blockers like TAK-242, can interrupt this lethal network axis at either the receptor or ligand level, effectively mitigating the severity of neurogenic ARDS and substantially increasing the overall survival rate of subjects with severe brain injury.

### 5.4 Holistic and non-invasive integrative modalities

Moving beyond acute pharmacological interventions, a holistic approach is essential for comprehensive brain-lung axis management. Emerging evidence highlights the relevance of the gut-lung-brain axis, where microbiome modulation through probiotics or targeted dietary interventions may offer systemic immunoregulatory benefits, attenuating both neuroinflammation and pulmonary immune hyper-reactivity (78–80). For patients with structural pre-morbid vulnerabilities, pulmonary-specific interventions can directly benefit glymphatic function. For instance, the use of continuous positive airway pressure (CPAP) in patients with pre-morbid OSA can restore normal sleep architecture and alleviate intermittent hypoxia, thereby rescuing circadian glymphatic clearance (81–83). Similarly, the application of senolytic therapy in COPD models has shown promise in clearing senescent cells, reducing systemic oxidative stress, and thereby ameliorating baseline neurovascular vulnerability (84–86). Furthermore, non-invasive integrative modalities present significant adjunct therapeutic value. Addressing the diaphragmatic role to improve inspiratory muscle function can optimize negative intrathoracic pressure mechanics, facilitating venous return and indirectly promoting CSF drainage. Additionally, alternative modalities such as acupuncture are gaining recognition for their potential to modulate neuroinflammation and balance autonomic dysregulation, offering a truly integrative pathway to stabilizing the bidirectional brain-lung axis (87–89).

### 5.5 Limitations and unresolved questions

Currently, most evidence is derived from murine models. Translating these findings to humans requires caution, as non-invasive, high-resolution imaging of the human glymphatic system remains technically challenging.

## 6 Conclusion and future directions

In summary, the pathological foundation of ARDS emerging as a fatal distal complication following acute brain injury is far deeper and more complex than the traditional concepts of “massive catecholamine release” and hydrostatic alterations. The latest molecular and anatomical evidence illustrates that the comprehensive severe impairment of the glymphatic system—the brain’s dedicated metabolic waste clearance network—is the keystone event triggering the collapse of the “brain-lung axis.” Mechanical stress and inflammatory responses cause the loss of AQP4 polarization, which, coupled with the anatomical spatial occlusion caused by astrogliosis, completely obstructs CSF-ISF convective exchange. This “pipeline failure” directly leads to a local accumulation of DAMPs (including HMGB1 and S100B) and pro-inflammatory cytokines within the brain parenchyma. Subsequently, these highly concentrated neurotoxins act like a breached dam, surging through the enzymatically degraded BBB or hijacking the inflammation-compromised meningeal lymphatics to flood the systemic circulation, either as free molecules or hidden within EVs. Upon reaching the pulmonary circulation, these mediators specifically bind and activate TLR4/RAGE receptors on endothelial cells, inducing downstream kinase cascades that cause the phosphorylation and internalization of VE-cadherin, completely obliterating the physical alveolar-capillary barrier. Simultaneously, they remodel the pulmonary immune microenvironment, driving alveolar macrophages toward M1 polarization and recruiting neutrophils to execute destructive NETosis, ultimately causing irreversible lung parenchymal damage and respiratory failure.

This precise mapping of network mechanisms offers revolutionary intervention targets for neurocritical care medicine. Looking ahead, translating these major laboratory findings into clinical life-saving capabilities will first require the use of high-resolution *in vivo* imaging technologies (such as Diffusion Tensor Image Analysis along the Perivascular Space, dynamic contrast-enhanced MRI, and PET tracers) to dynamically monitor glymphatic stagnation and its temporal correlation with pulmonary functional deterioration in both live models and humans (10). Moreover, deep profiling of the specific molecular cargo (e.g., miRNA transcriptomes) of brain-derived EVs in plasma holds promise for providing exquisitely sensitive liquid biopsy biomarkers for early warning of brain-lung injury (10). Therapeutically, a multidimensional combination strategy aimed at disrupting the bidirectional vicious cycle of the brain-lung axis—integrating Dexmedetomidine to simulate sleep and protect AQP4 polarization, gene modification with VEGF-C to selectively promote meningeal lymphatic drainage, and anti-HMGB1 antibodies to cast an immunological safety net in the peripheral circulation—will undoubtedly offer the most promising medical breakthrough to shatter the malignant pathological loop of the “brain-lung axis” and save the lives of patients with severe brain injuries.

## References

[1] Mrozek S Constantin JM Geeraerts T. Brain-lung crosstalk: implications for neurocritical care patients. World J Crit Care Med. (2015) 4:163–78. doi: 10.5492/wjccm.v4.i3.163, 26261769 PMC4524814

[2] Matin N Sarhadi K Crooks CP Lele AV Srinivasan V Johnson NJ . Brain-lung crosstalk: Management of Concomitant Severe Acute Brain Injury and Acute Respiratory Distress Syndrome. Curr Treat Options Neurol. (2022) 24:383–408. doi: 10.1007/s11940-022-00726-3, 35965956 PMC9363869

[3] Song M Jung S Go KO Lee K Heo W Hwang SH . Clinical outcomes of extracorporeal membrane oxygenation for acute respiratory distress syndrome in patients with traumatic brain injury or spontaneous intracerebral and subarachnoid hemorrhages: a retrospective PILOT study. Medicina (Kaunas). (2025) 62:13. doi: 10.3390/medicina62010013, 41597299 PMC12842940

[4] Aisiku IP Yamal JM Doshi P Rubin ML Benoit JS Hannay J . The incidence of ARDS and associated mortality in severe TBI using the Berlin definition. J Trauma Acute Care Surg. (2016) 80:308–12. doi: 10.1097/TA.0000000000000903, 26491799 PMC4731296

[5] Hendrickson CM Howard BM Kornblith LZ Conroy AS Nelson MF Zhuo H . The acute respiratory distress syndrome following isolated severe traumatic brain injury. J Trauma Acute Care Surg. (2016) 80:989–97. doi: 10.1097/TA.0000000000000982, 26881489 PMC5851280

[6] Baumann A Audibert G McDonnell J Mertes PM. Neurogenic pulmonary edema. Acta Anaesthesiol Scand. (2007) 51:447–55. doi: 10.1111/j.1399-6576.2007.01276.x, 17378783

[7] Finsterer J. Neurological perspectives of neurogenic pulmonary edema. Eur Neurol. (2019) 81:94–102. doi: 10.1159/000500139, 31117074

[8] Davison DL Terek M Chawla LS. Neurogenic pulmonary edema. Crit Care. (2012) 16:212. doi: 10.1186/cc11226, 22429697 PMC3681357

[9] Thompson BT Chambers RC Liu KD. Acute respiratory distress syndrome. N Engl J Med. (2017) 377:562–72. doi: 10.1056/NEJMra1608077, 28792873

[10] Jessen NA Munk ASF Lundgaard I Nedergaard M. The Glymphatic system: a beginner's guide. Neurochem Res. (2015) 40:2583–99. doi: 10.1007/s11064-015-1581-6, 25947369 PMC4636982

[11] Mestre H Hablitz LM Xavier ALR Feng W Zou W Pu T . Aquaporin-4-dependent glymphatic solute transport in the rodent brain. eLife. (2018) 7:40070. doi: 10.7554/eLife.40070, 30561329 PMC6307855

[12] Amiry-Moghaddam M Williamson A Palomba M Eid T de Lanerolle NC Nagelhus EA . Delayed K+ clearance associated with aquaporin-4 mislocalization: phenotypic defects in brains of alpha-syntrophin-null mice. Proc Natl Acad Sci USA. (2003) 100:13615–20. doi: 10.1073/pnas.2336064100, 14597704 PMC263862

[13] Iliff JJ Wang M Liao Y Plogg BA Peng W Gundersen GA . A paravascular pathway facilitates CSF flow through the brain parenchyma and the clearance of interstitial solutes, including amyloid β. Sci Transl Med. (2012) 4:3748. doi: 10.1126/scitranslmed.3003748, 22896675 PMC3551275

[14] Zahran A Abu-Khazneh O Bdair M Hajjeh O AbuBaha M Shehadeh W . Glymphatic system dysfunction in central nervous system diseases. CNS Neurosci Ther. (2026) 32:e70810. doi: 10.1002/cns.70810, 41792880 PMC12965907

[15] Hwang EH Koo JH Lee YH Song JH Lim YC. Neurogenic pulmonary edema and Takotsubo cardiomyopathy in aneurysmal subarachnoid hemorrhage. Acta Neurochir. (2023) 165:3677–84. doi: 10.1007/s00701-023-05824-y, 37924360

[16] Johnson NH Casanova NG Patarroyo-White S Canizales J Camp SM Barcena JP . Lung-brain Axis-generated inflammatory biomarkers in traumatic brain injury and acute respiratory distress syndrome: role of mechanical ventilation/stress. Adv Biomark Sci Technol. (2025) 7:238–47. doi: 10.1016/j.abst.2025.08.002, 41497388 PMC12768522

[17] Wu J Gao W Zhang H. Development of acute lung injury or acute respiratory distress syndrome after subarachnoid hemorrhage, predictive factors, and impact on prognosis. Acta Neurol Belg. (2023) 123:1331–7. doi: 10.1007/s13760-023-02207-z, 36922484 PMC10338383

[18] Cândido LDS Lopes MR Machado-Junior PA dos Santos AM di Paschoale Ostolin TLV de Souza ABF . Impact of mechanical ventilation on oxidative stress, neuroinflammation, memory and anxiety related behavior: insights from an experimental model. Respir Physiol Neurobiol. (2026) 342:104565. doi: 10.1016/j.resp.2026.104565, 41850582

[19] Han S Wang Z Zheng Q Guo F Xiao Y Wang Z . Sympathetic Overactivation drives neurogenic alveolar epithelial Pyroptosis via the PIEZO2-ER stress pathway in acute lung injury following intracerebral hemorrhage. CNS Neurosci Ther. (2026) 32:e71010. doi: 10.1002/cns.71010, 42405383 PMC13334374

[20] Wu D Liao X Gao J Wang M Meng L Xu W . Dopamine signaling governs macrophage-mediated acute lung injury through JAML/IL-10-coupled mitochondrial regulation. J Neuroinflammation. (2026) 23:195. doi: 10.1186/s12974-026-03823-1, 42026623 PMC13248467

[21] Sharie SA Almari R Azzam S al-Husinat L Araydah M Battaglini D . Brain protective ventilation strategies in severe acute brain injury. Curr Neurol Neurosci Rep. (2025) 25:68. doi: 10.1007/s11910-025-01462-2, 41082009 PMC12518427

[22] Slutsky AS Ranieri VM. Ventilator-induced lung injury. N Engl J Med. (2013) 369:2126–36. doi: 10.1056/NEJMra1208707, 24283226

[23] Xie L Kang H Xu Q Chen MJ Liao Y Thiyagarajan M . Sleep drives metabolite clearance from the adult brain. Science. (2013) 342:373–7. doi: 10.1126/science.1241224, 24136970 PMC3880190

[24] Plog BA Dashnaw ML Hitomi E Peng W Liao Y Lou N . Biomarkers of traumatic injury are transported from brain to blood via the glymphatic system. J Neurosci. (2015) 35:518–26. doi: 10.1523/JNEUROSCI.3742-14.2015, 25589747 PMC4293408

[25] Wardlaw JM Benveniste H Nedergaard M Zlokovic BV Mestre H Lee H . Perivascular spaces in the brain: anatomy, physiology and pathology. Nat Rev Neurol. (2020) 16:137–53. doi: 10.1038/s41582-020-0312-z, 32094487

[26] Kitchen P Salman MM Halsey AM Clarke-Bland C MacDonald JA Ishida H . Targeting Aquaporin-4 subcellular localization to treat central nervous system edema. Cell. (2020) 181:784–799.e19. doi: 10.1016/j.cell.2020.03.037, 32413299 PMC7242911

[27] Tourdias T Mori N Dragonu I Cassagno N Boiziau C Aussudre J . Differential aquaporin 4 expression during edema build-up and resolution phases of brain inflammation. J Neuroinflammation. (2011) 8:143. doi: 10.1186/1742-2094-8-143, 22011386 PMC3220647

[28] Rossi A Moritz TJ Ratelade J Verkman AS. Super-resolution imaging of aquaporin-4 orthogonal arrays of particles in cell membranes. J Cell Sci. (2012) 125:109603. doi: 10.1242/jcs.109603, 22718347 PMC3516444

[29] Iliff JJ Chen MJ Plog BA Zeppenfeld DM Soltero M Yang L . Impairment of glymphatic pathway function promotes tau pathology after traumatic brain injury. J Neurosci. (2014) 34:16180–93. doi: 10.1523/JNEUROSCI.3020-14.2014, 25471560 PMC4252540

[30] Kress BT Iliff JJ Xia M Wang M Wei HS Zeppenfeld D . Impairment of paravascular clearance pathways in the aging brain. Ann Neurol. (2014) 76:845–61. doi: 10.1002/ana.24271, 25204284 PMC4245362

[31] Broomand Lomer N Ahmadzadeh AM Ashoobi MA Diaz-Arrastia R Verma R. Glymphatic system dysfunction and diffusion tensor imaging along the perivascular space in traumatic brain injury: a systematic review and meta-analysis. AJNR Am J Neuroradiol. (2026) 47:2167–861. doi: 10.3174/ajnr.A9223 PMC13431558 41667226

[32] Fang J Pan X Chen J Yang X Wu Z Ying Z . Aquaporin 4 knockdown alleviates traumatic brain edema and reduces neuronal axonal growth cone collapse via the RhoA/ROCK pathway. Neurobiol Dis. (2026) 223:107390. doi: 10.1016/j.nbd.2026.107390, 41967652

[33] Chen Y Yin X Zhang E Li B Yu H Qi J . Astrocyte-derived Exosomal miR-211-5p alleviates blood-brain barrier injury in a rat model of traumatic brain injury. CNS Neurosci Ther. (2026) 32:e70858. doi: 10.1002/cns.70858, 41954515 PMC13064413

[34] Shen W Jiang C Liu X Wang D Li G Hui Y . Astragaloside IV alleviates post-traumatic cytotoxic edema via inhibition of AQP4 expression and subcellular localization. Phytomedicine. (2026) 155:158096. doi: 10.1016/j.phymed.2026.158096, 41916121

[35] Ouyang Q Yang Y Wang J Liang A Wang K Yuan Y . C-Src/β-DG-mediated impairment of glia-vascular unit integrity via AQP4 depolarization contributes to secondary hydrocephalus after intraventricular hemorrhage. Int J Surg. (2026) 112:3028–41. doi: 10.1097/JS9.0000000000003867, 41208590

[36] Oka F Yamane M Kawano R Nishimoto T Mori N Kawano A . MRI-based ALPS index as a biomarker of Glymphatic dysfunction and prognosis after aneurysmal subarachnoid hemorrhage. Stroke Vasc Interv Neurol. (2026) 6:e001903. doi: 10.1161/SVIN.125.001903, 41815303 PMC12959432

[37] Jin L Yang Z Wei B Wu Y Li L Zhou J . Amelioration of post-stroke edema and microcirculatory dysfunction via targeted AQP4 inhibition while preserving the glymphatic system. Adv Sci (Weinh). (2026) 13:e20118. doi: 10.1002/advs.202520118, 41387988 PMC12915113

[38] Paudel YN Angelopoulou E Piperi C Othman I Shaikh MF. HMGB1-mediated Neuroinflammatory responses in brain injuries: potential mechanisms and therapeutic opportunities. Int J Mol Sci. (2020) 21:609. doi: 10.3390/ijms21134609, 32610502 PMC7370155

[39] Bhandari B Naeini SE Rogers HM Alhashim AH Yu JC Seyyedi M . Protective role of CBD against nicotine pouch-induced seizure aggravation and alterations in brain Glymphatic biomarkers. Nicotine Tob Res. (2026) 28:848–56. doi: 10.1093/ntr/ntaf253, 41384771 PMC13101982

[40] Zhu X Lin J Yang P Wu S Lin H He W . Surgery induces neurocognitive disorder via neuroinflammation and glymphatic dysfunction in middle-aged mice with brain lymphatic drainage impairment. Front Neurosci. (2024) 18:1426718. doi: 10.3389/fnins.2024.1426718, 38975244 PMC11225229

[41] Murcko R Marchi N Bailey D Janigro D. Diagnostic biomarker kinetics: how brain-derived biomarkers distribute through the human body, and how this affects their diagnostic significance: the case of S100B. Fluids Barriers CNS. (2022) 19:32. doi: 10.1186/s12987-022-00329-9, 35546671 PMC9092835

[42] Lindblad C Nelson DW Zeiler FA Ercole A Ghatan PH von Horn H . Influence of blood-brain barrier integrity on brain protein biomarker clearance in severe traumatic brain injury: a longitudinal prospective study. J Neurotrauma. (2020) 37:1381–91. doi: 10.1089/neu.2019.6741, 32013731 PMC7249468

[43] Schob S Puchta J Winter K Michalski D Mages B Martens H . Surfactant protein C is associated with perineuronal nets and shows age-dependent changes of brain content and hippocampal deposits in wildtype and 3xTg mice. J Chem Neuroanat. (2021) 118:102036. doi: 10.1016/j.jchemneu.2021.102036, 34626771

[44] Ferrara M Bertozzi G Volonnino G di Fazio N Frati P Cipolloni L . Glymphatic system a window on TBI pathophysiology: a systematic review. Int J Mol Sci. (2022) 23:138. doi: 10.3390/ijms23169138, 36012401 PMC9408940

[45] Fei X Chen C Kai S Fu X Man W Ding B . Eupatilin attenuates the inflammatory response induced by intracerebral hemorrhage through the TLR4/MyD88 pathway. Int Immunopharmacol. (2019) 76:105837. doi: 10.1016/j.intimp.2019.105837, 31476693

[46] Xu H He J du H Jing X Liu X. Evaluation of the choroid plexus epithelium inflammation TLR4/NF-κB/NKCC1 signal pathway activation in the development of hydrocephalus. CNS Neurosci Ther. (2024) 30:e70085. doi: 10.1111/cns.70085, 39450988 PMC11503839

[47] Xu N Li X Weng J Wei C He Z Doycheva DM . Adiponectin ameliorates GMH-induced brain injury by regulating microglia M1/M2 polarization via AdipoR1/APPL1/AMPK/PPARγ signaling pathway in neonatal rats. Front Immunol. (2022) 13:873382. doi: 10.3389/fimmu.2022.873382, 35720361 PMC9203698

[48] Tan L Liang J Wang X Wang Y Xiong T. Dual roles of microglia in the pathological injury and repair of hemorrhagic cerebrovascular diseases. Regen Med Rep. (2024) 1:93–105. doi: 10.4103/RMR.REGENMED-D-24-00001

[49] Wang J Lei Y Yang Y Wang J. Meningeal lymphatics drives macrophage clearance via CCL2-CCR2 Axis after cerebral ischemia. Curr Issues Mol Biol. (2026) 48:259. doi: 10.3390/cimb48030259, 41899411 PMC13024750

[50] Wang CL Tseng GF Tsai ST Chen LJ. Intracranial inflammation and meningeal fibrosis are associated with perivascular changes, altered CSF tracer dynamics, and cognitive decline in a rat model of communicating hydrocephalus. Fluids Barriers CNS. (2026) 23:40. doi: 10.1186/s12987-026-00787-5, 41787552 PMC12964795

[51] Zhang Q Chen Y Li Y Feng Z Liang L Hao X . Neutrophil extracellular trap-mediated impairment of meningeal lymphatic drainage exacerbates secondary hydrocephalus after intraventricular hemorrhage. Theranostics. (2024) 14:1909–38. doi: 10.7150/thno.91653, 38505607 PMC10945341

[52] Yang Y Ouyang Q Sun Y Liang A Wang J Wang K . Therapeutic hypothermia alleviates hydrocephalus and neurological dysfunction post intraventricular hemorrhage by enhancing drainage of Glymphatic-meningeal lymphatic-deep cervical lymphatic system. CNS Neurosci Ther. (2026) 32:e70740. doi: 10.1002/cns.70740, 41578884 PMC12831171

[53] Chen W Wu ZC Lin L Huang S Jiang Q Huang H . Effect of intranasal treatment with NAMPT-EVs on acetylated tau and cognitive function in mice with repeated controlled cortical injury. Int Immunopharmacol. (2026) 175:116415. doi: 10.1016/j.intimp.2026.116415, 41747594

[54] Zhang Y Sun C Wang B Gu A Zhou Z Gu C. Brain-derived Exosomal miR-9-5p induces Ferroptosis in traumatic brain injury-induced acute lung injury by targeting Scd1. CNS Neurosci Ther. (2024) 30:e70189. doi: 10.1111/cns.70189, 39723576 PMC11669946

[55] Kerr NA de Rivero Vaccari JP Abbassi S Kaur H Zambrano R Wu S . Traumatic brain injury-induced acute lung injury: evidence for activation and inhibition of a neural-respiratory-Inflammasome Axis. J Neurotrauma. (2018) 35:2067–76. doi: 10.1089/neu.2017.5430, 29648974 PMC6098413

[56] Johnston W Arzave J Yuengert C Park DJ Costantini TW Eliceiri BP . TBI-associated acute lung injury is associated with downregulation of miRNA-362. J Trauma Acute Care Surg. (2026) 100:779–86. doi: 10.1097/TA.0000000000004937, 42030095

[57] Sun Y Shu X Yuan H Qin B Guo F. Ferroptosis, orchestrated by GPX4 downregulation, serves as a critical mediator of neutrophil extracellular trap-driven pathology in hypoxic pulmonary edema. Apoptosis. (2026) 31:140. doi: 10.1007/s10495-026-02331-0, 42115527

[58] Li C Gao P Zhuang F Wang T Wang Z Wu G . Inhibition of ALOX12-12-HETE alleviates lung ischemia-reperfusion injury by reducing endothelial ferroptosis-mediated neutrophil extracellular trap formation. Research (Wash D C). (2024) 7:0473. doi: 10.34133/research.0473 39268501 PMC11391482

[59] Zhao MJ Jiang HR Sun JW Wang ZA Hu B Zhu CR . Roles of RAGE/ROCK1 pathway in HMGB1-induced early changes in barrier permeability of human pulmonary microvascular endothelial cell. Front Immunol. (2021) 12:697071. doi: 10.3389/fimmu.2021.697071, 34745088 PMC8564108

[60] Wang Y Zhu X Li L Su D Ai L Xie H . Fibroblast EGFR signaling mediates ricin toxin-induced acute lung injury via EGR1/CXCL1 axis. Arch Toxicol. (2025) 99:3413–27. doi: 10.1007/s00204-025-04067-3, 40317338

[61] Papayannopoulos V. Neutrophil extracellular traps in immunity and disease. Nat Rev Immunol. (2018) 18:134–47. doi: 10.1038/nri.2017.105, 28990587

[62] Humayun M Premraj L Shah V Cho SM. Mechanical ventilation in acute brain injury patients with acute respiratory distress syndrome. Front Med (Lausanne). (2022) 9:999885. doi: 10.3389/fmed.2022.999885, 36275802 PMC9582443

[63] Huang M Gedansky A Hassett CE Price C Fan TH Stephens RS . Pathophysiology of brain injury and neurological outcome in acute respiratory distress syndrome: a scoping review of preclinical to clinical studies. Neurocrit Care. (2021) 35:518–27. doi: 10.1007/s12028-021-01309-x, 34297332 PMC8299740

[64] Ma T Liu J Gao Z Li Y Lin J Pang K . Bone marrow-derived emergency monopoiesis drives brain-lung axis injury after traumatic brain injury via IL-1. J Neuroinflammation. (2025) 22:262. doi: 10.1186/s12974-025-03589-y, 41194087 PMC12590780

[65] Zou Z Liu Y Lu Y Deng W Huang Q Li L . Crosstalk between astrocytes and neutrophils via S100B/RAGE/NETs exacerbates secondary injury following traumatic brain injury. Brain Behav Immun. (2026) 131:106153. doi: 10.1016/j.bbi.2025.106153, 41138886

[66] Ghaderi S Mohammadi S Fatehi F. Glymphatic pathway dysfunction in severe obstructive sleep apnea: a meta-analysis. Sleep Med. (2025) 131:106528. doi: 10.1016/j.sleep.2025.106528, 40267528

[67] Ke S Luo T Ding Y Tang CJ Jie Z Shen JZ . Does obstructive sleep apnea mediate the risk of cognitive impairment by expanding the perivascular space? Sleep Breath. (2025) 29:130. doi: 10.1007/s11325-025-03291-6, 40085157

[68] Peng C Sun X Zhang Z Jia M Jin J He Y . Chronic intermittent hypoxia impairs glymphatic function in male mice through ENT-dependent adenosine dysregulation. Sleep. (2026):zsag182. doi: 10.1093/sleep/zsag182, 42412752

[69] Sun X Fang D Zhang X Zhu H Sun Y Liu G . Engineering near-infrared-II nanoprobes reveal dexmedetomidine potentiating brain waste clearance in healthy and sleep-restricted mice. ACS Nano. (2025) 19:34830–46. doi: 10.1021/acsnano.5c10519 41004722

[70] Wang S Yu X Cheng L Ren W Wen G Wu X . Dexmedetomidine improves the circulatory dysfunction of the glymphatic system induced by sevoflurane through the PI3K/AKT/ΔFosB/AQP4 pathway in young mice. Cell Death Dis. (2024) 15:448. doi: 10.1038/s41419-024-06845-w, 38918408 PMC11199640

[71] Sun B Fang D Li W Li M Zhu S. NIR-II nanoprobes for investigating the glymphatic system function under anesthesia and stroke injury. J Nanobiotechnology. (2024) 22:200. doi: 10.1186/s12951-024-02481-w, 38654299 PMC11040925

[72] Igarashi H Huber VJ Tsujita M Nakada T. Pretreatment with a novel aquaporin 4 inhibitor, TGN-020, significantly reduces ischemic cerebral edema. Neurol Sci. (2011) 32:113–6. doi: 10.1007/s10072-010-0431-1, 20924629 PMC3026762

[73] Giannetto MJ Gomolka RS Gahn‐Martinez D Newbold EJ Bork PAR Chang E . Glymphatic fluid transport is suppressed by the aquaporin-4 inhibitor AER-271. Glia. (2024) 72:982–98. doi: 10.1002/glia.24515, 38363040 PMC11203403

[74] Liao J Duan Y Liu Y Chen H An Z Chen Y . Simvastatin alleviates glymphatic system damage via the VEGF-C/VEGFR3/PI3K-Akt pathway after experimental intracerebral hemorrhage. Brain Res Bull. (2024) 216:111045. doi: 10.1016/j.brainresbull.2024.111045, 39097032

[75] Li MN Jing YH Wu C Li X Liang FY Li G . Continuous theta burst stimulation dilates meningeal lymphatic vessels by up-regulating VEGF-C in meninges. Neurosci Lett. (2020) 735:135197. doi: 10.1016/j.neulet.2020.135197, 32590044

[76] Song E Mao T Dong H Boisserand LSB Antila S Bosenberg M . VEGF-C-driven lymphatic drainage enables immunosurveillance of brain tumours. Nature. (2020) 577:689–94. doi: 10.1038/s41586-019-1912-x, 31942068 PMC7100608

[77] Keuters MH Antila S Immonen R Plotnikova L Wojciechowski S Lehtonen S . The impact of VEGF-C-induced Dural lymphatic vessel growth on ischemic stroke pathology. Transl Stroke Res. (2025) 16:781–99. doi: 10.1007/s12975-024-01262-9, 38822994 PMC12045824

[78] Mathias K Petronilho F Danielski LG. Nose-to-brain axis: mechanistic links between nasal microbiome dysbiosis, neuroinflammation, and brain disorders. Neuroscience. (2026) 598:19–28. doi: 10.1016/j.neuroscience.2026.01.039, 41619875

[79] Liu A Zhang XQ Guo JX Wen QY Dai K Zheng WJ . Esketamine alleviates COPD-depression comorbidity in rats via MAPK/NF-κB inhibition and gut-lung-brain axis modulation. J Neuroinflammation. (2026) 23:68. doi: 10.1186/s12974-026-03699-1, 41572340 PMC12911015

[80] Cheng Y Hu G Deng L Zan Y Chen X. Therapeutic role of gut microbiota in lung injury-related cognitive impairment. Front Nutr. (2024) 11:1521214. doi: 10.3389/fnut.2024.1521214, 40017811 PMC11867030

[81] Sommer R Hill EA Ramirez J Coello RD Ballerini L Gibson E . Continuous positive airway pressure and progression of enlarged perivascular spaces in adults with obstructive sleep apnea. Neurology. (2025) 105:e213955. doi: 10.1212/WNL.0000000000213955, 41021868

[82] Fernandes M Placidi F Izzi F Nuccetelli M Bernardini S Mercuri NB . Persistent blood-brain barrier dysregulation in patients with obstructive sleep apnea following long-term continuous positive airway pressure treatment. Neurobiol Aging. (2025) 151:89–94. doi: 10.1016/j.neurobiolaging.2025.04.001, 40267730

[83] Lee HJ. Could positive airway pressure enhance brain waste clearance and modify neurodegenerative risk? A perspective on sleep-dependent cerebrospinal fluid-lymphatic pathways. Sleep. (2026) 49:zsag065. doi: 10.1093/sleep/zsag065, 41786635 PMC13163175

[84] van der Koog L Showell HRD Nugraha DF Lehmann M Conlon TM Yildirim AÖ . Regenerative therapeutics for chronic obstructive pulmonary disease. Pharmacol Rev. (2026) 78:100124. doi: 10.1016/j.pharmr.2026.100124, 41856010 PMC13197932

[85] Hao J Liu Z Zhi Y Lu L Xu X Lu F . Novel therapeutic targets and drug discovery strategies in chronic obstructive pulmonary disease. Pharmacol Ther. (2026) 284:109040. doi: 10.1016/j.pharmthera.2026.109040, 42066848

[86] Liao S Chen D Long H Jiang S Fan J Li S . Hydrogen sulfide attenuates oxidative stress-induced cellular senescence via the Sirt3/SOD2 signaling pathway in chronic obstructive pulmonary disease. Chin Med J. (2026) 139:866–79. doi: 10.1097/CM9.0000000000003452, 40082252 PMC12999109

[87] Ba T Niu R Gao J Li W Dong M Duan J . Microbiota-gut-brain-axis: a new target of acupuncture therapy for post-stroke cognitive impairment. Front Microbiol. (2025) 16:1425054. doi: 10.3389/fmicb.2025.1425054, 40342596 PMC12058749

[88] Zhu J Ge X Cao Y Xiao R Deng X. Neuroprotective effects of electroacupuncture in ischemic stroke: from mechanisms to clinical implications. Front Aging Neurosci. (2025) 17:1562925. doi: 10.3389/fnagi.2025.1562925, 40353059 PMC12063359

[89] Yang G Zhang L Yuan Y Mazhar M Zhang D Liu Y . Electroacupuncture improves microglial polarization induced-inflammation by regulating the TGF-β/Smad-3 signaling pathway in ischemic stroke mice. CNS Neurosci Ther. (2025) 31:e70567. doi: 10.1111/cns.70567, 40831324 PMC12365391

